# Fabrication of nickel phytate modified bio-based polyol rigid polyurethane foam with enhanced compression-resistant and improved flame-retardant

**DOI:** 10.1038/s41598-024-67520-w

**Published:** 2024-07-19

**Authors:** Xu Zhang, Zhaoqian Wang, Shuai Ding, Zhi Wang

**Affiliations:** 1 Liaoning Key Laboratory of Aircraft Fire Explosion Control and Reliability Airworthiness Technology, Shenyang Aerospace University, Shenyang, 110136 China; 2 School of Safety Engineering, Shenyang Aerospace University, Shenyang, 110136 China

**Keywords:** Rigid polyurethane foam, nickel phytate, Thermal stability, Flame retardant, Compression resistant, Polymers, Mechanical properties, Characterization and analytical techniques

## Abstract

A bio-based flame retardant nickel phytate (PA-Ni) was synthesized and combined with soybean oil-based polyol (SO) to create a green rigid polyurethane foam (RPUF) with enhanced compressive strength, good thermal stability and flame retardant. The results showed that the RPUF-SO2/Ni3 with 3 wt% PA-Ni had the highest initial and termination temperature, maximum thermal decomposition rate temperature and carbon residue, and better thermal stability. Its limiting oxygen index was increased by 2.6% compared with RPUF-SO2 without PA-Ni added, and the peak heat release rate (PHRR) and total heat release rate (THR) were reduced by 14.92% and 19.92%, respectively. In addition, RPUF-SO2/Ni3 had the lowest Ds under the conditions of flame (18.90) and flameless (22.41), and had the best smoke suppression effect. And the compressive strength of RPUF-SO/Ni3 was significantly enhanced by the addition of PA-Ni. The results show that the improvement of flame retardancy of RPUF is mainly the result of the combined effect of gas-phase and condensed-phase flame retardancy, among which the flame retardancy of RPUF-SO/Ni3 was the best. The current findings offer a practical way to produce green and low-carbon RPUF as well as promising prospects for the material's safe application.

## Introduction

Rigid polyurethane foam (RPUF) is a very important polymer material, which is made of polyether polyol, isocyanuric acid, foaming, crosslinker, etc. as the main raw materials^1,2^. The RPUF is widely used in aviation, shipbuilding, construction, pipeline and other industries due to its small apparent density, good mechanical properties and light weight^3–5^. However, the RPUF has a low limiting oxygen index (LOI), is flammable, and will produce a large amount of toxic smoke during combustion, which seriously endangers human health^6,7^. Therefore, it is necessary to prepare the RPUF with excellent flame retardant properties.

Halogenated flame retardants were once widely used for flame-retardant modification of the RPUF, but they released substances that are harmful to human beings during the combustion process, so the use of such materials has been strictly controlled by society and regulatory authorities^8^. In addition, the exploration and use of non-halogenated biomass-based flame retardants has attracted widespread attention as the concept of "green, low-carbon and sustainable" is becoming increasingly popular. Among them, the phosphorus-based flame retardants represented by bio-based phytic acid (PA) are the most representative. PA is derived from plant stalks and is abundant in nature, which can alleviate the shortage of non-renewable energy^9^. In addition, due to its special self-assembled structure, it is not only rich in phosphorus, but also can form complexes with metal ions to enhance its flame retardant properties.

Yu et al. first reacted PA with hydrolyzed silk (HS) to obtain the product PA@HS, and then compounded it with potato starch (PS) to prepare a biomass intumescent flame retardant material with good flame retardant properties, and blended it with polyacrylonitrile (PAN) to obtain flame retardant^10^. It was found that compared with the control group, the peak heat release rate (PHRR) and the peak smoke production rate (PSPR) of PAN-10% PA@HS/5% decreased by 54.8% and 79.3%, which had better smoke suppression. Liu et al. introduced PA, serine (SE) and vitamin B2 (VB2) into polyacrylonitrile (PAN) as raw materials to prepare new flame retardants with good performance^11^. Compared with the original sample, the PHRR and total smoke production (TSP) of PAN-10%PA@SE/5% VB2 were reduced by 57.4% and 65.4%, respectively, which effectively improved its fire resistance. Gao et al. reported the salt-forming reaction of PA with piperazine, and prepared a novel phytate PHYPI, which was used for flame retardant polypropylene (PP)^12^. The limiting oxygen index (LOI) value of PP with 18.0 wt% PHYPI loading was 25.0%, which was 38.9% higher than that of blank PP. Zhang et al. successfully prepared manganese phytate (MnPa) and compounded it with expandable graphite (EG) to prepare MEPUF2 (MnPa/EG = 1/1) with the highest activation energy, and its light transmittance was increased by 5.1% compared with MEPUF1 (MnPa/EG = 3/1)^13^. Its heat resistance and smoke suppression properties were also improved. Zhang et al. used PA and barium carbonate to prepare barium phytate (PABA) for flame retardant modification of the RPUF^14^. The results showed that the addition of PABA improved the flame retardancy of the RPUF, the PHRR (44.69 kW/m^2^) and total heat release (THR, 2.49 MJ/m^2^) of 15% PABA (FPUF-PABA15) were the lowest among all products.

In order to explore the preparation of a green, low-carbon and low-toxicity bio-based RPUF, the current work chose PA and nickel acetate tetrahydrate (Ni(Ac)_2_·4H_2_O) as the base materials to prepare a biomass flame retardant (PA-Ni) and form a flame-retardant system with soybean oil-based polyols for flame retardant and smoke inhibition treatment of the RPUF. The thermal stability, flame retardancy, smoke toxicity and mechanical properties of the modified RPUFs were thoroughly investigated by some typical characterization methods, and the corresponding flame retardant mechanisms were proposed.

## Experiments

### Material

Soybean oil polyol (SO, hydroxyl value: 450 ± 30 KOH/g) was provided by Baichuan Chemical Co., Ltd. (Jining, China), polyether polyol (3630), triethanolamine, silicone oil (AK-8805) and stannous octanoate (T9) were purchased from Zhuolian Zhichuang Polymer Co., Ltd. (Changzhou, China), polyisocyanate methylene (PM200) was purchased from Wanhua Polyurethane Co., Ltd. (Yantai, China), phytic acid (PA, ≥ 70%), 95% The absolute ethanol was provided by Sinopharm Chemical Co., Ltd. (China), the nickel acetate tetrahydrate (Ni(Ac)_2_·4H_2_O, analytically pure) was provided by Tianjin Huasheng Chemical Reagent Co., Ltd. (Tianjin, China), and the deionized water was prepared by this laboratory.

### Synthesis of PA-Ni

**Figure 1:**
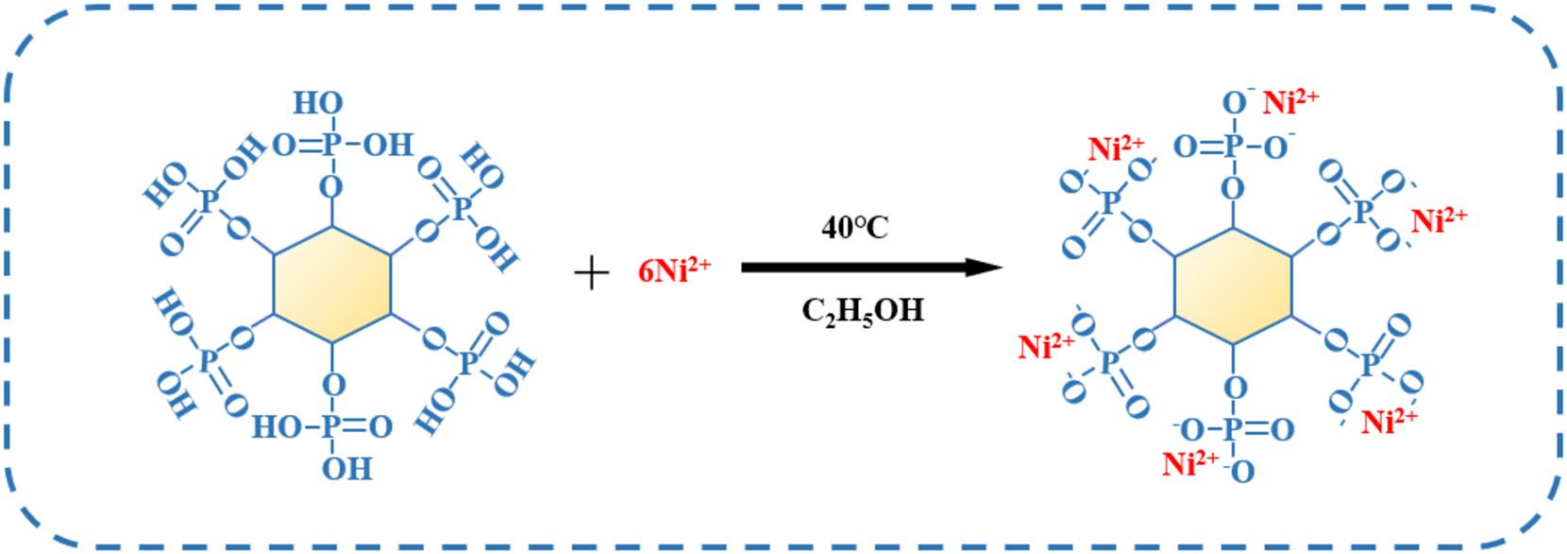
Synthesis mechanism of PA-Ni.

First, 70% PA solution (0.005 mol, 4.72 g) was dissolved in 50 ml of ethanol, and Ni(Ac)_2_·4H_2_O (0.03 mol, 7. 45 g) was dissolved in a volume of 50 ml of ethanol at a temperature of 40 °C and stirred to dissolve. Then the PA solution was dropped into this solution, which was stirred to make the solution mixed, and continued the reaction for 2 h. A light green precipitate was obtained, washed with anhydrous ethanol, and the precipitate was dried in a drying oven to obtain a light green powder, which was the flame retardant PA-Ni. The synthetic reaction was shown in Fig. 1.

### Synthesis of RPUF-SO/Nix composites

**Figure 2:**
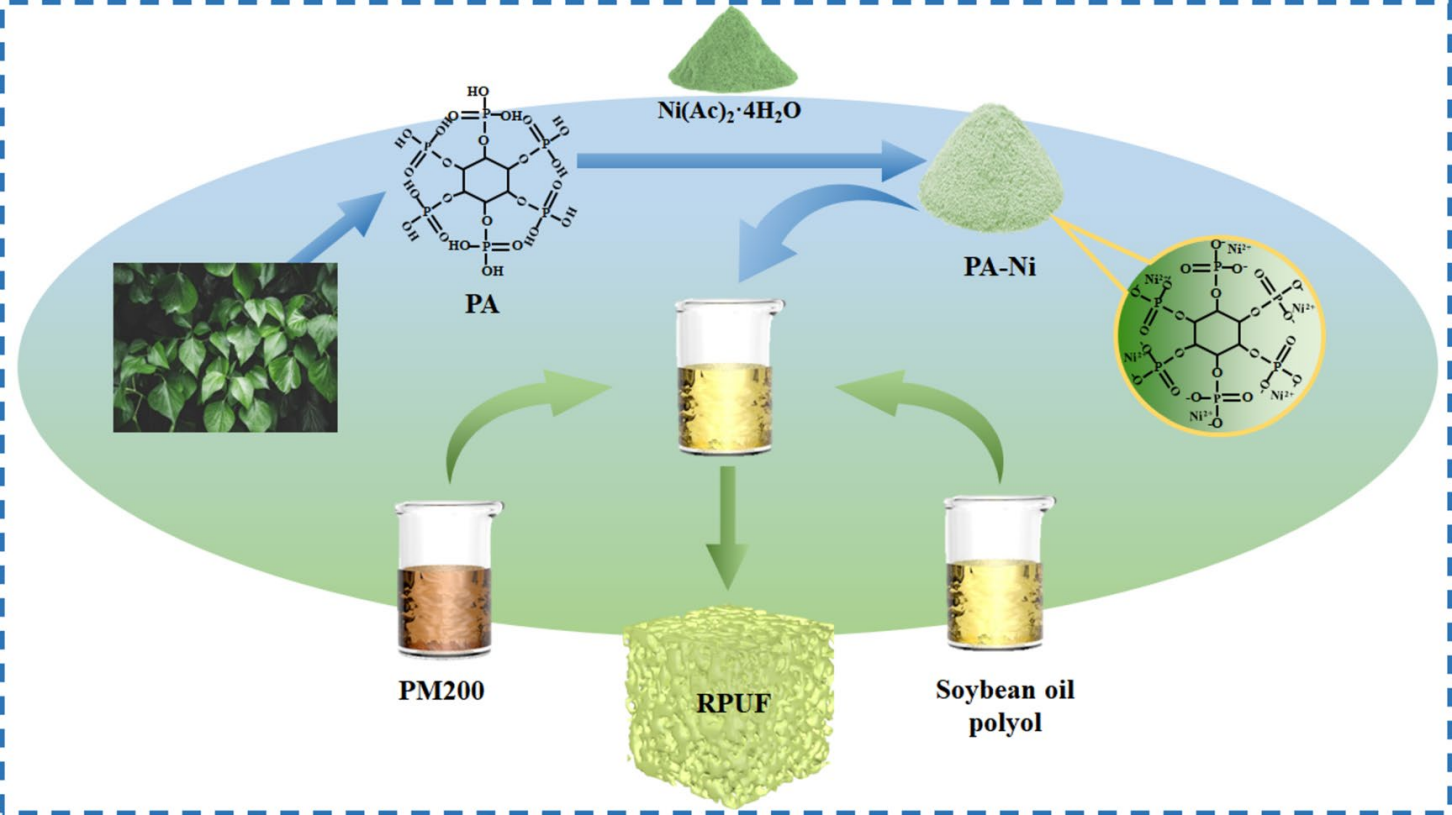
Preparation process of the modified RPUFs.

The RPUFs were prepared according to the formulation shown in Table 1. The production process was shown in Fig. 2. First of all, the A component (SO polyol, 3630 polyol, triethanolamine, AK-8805, T9, deionized water, PA-Ni) and B component (PM 200) were mixed in two different paper cups, and then the B solution was quickly poured into the A mixture, and stirred vigorously for about 50 s. The mixture was quickly poured into the open mold and finally foamed freely at room temperature for 24 h. All products obtained were denoted as RPUF-SO2/Nix (x = 1, 2, 3, 4, 5), where x represents the proportion of PA-Ni in the RPUF (wt%). RPUF-SO2 was set as the control group.

**Table 1:** Raw material formulation of the modified RPUFs.

Sample	Polyether polyol 3630N (g)	PM200 (g)	T9 (g)	AK-8805 (g)	Triethanolamine (g)	Deionized water (g)	SO polyol (wt%)	PA-Ni (wt%)
RPUF-SO2/Ni1	40	60	0.2	1	1.8	1	10	1
RPUF-SO2/Ni2	40	60	0.2	1	1.8	1	10	2
RPUF-SO2/Ni3	40	60	0.2	1	1.8	1	10	3
RPUF-SO2/Ni4	40	60	0.2	1	1.8	1	10	4
RPUF-SO2/Ni5	40	60	0.2	1	1.8	1	10	5

### Characterization

The prepared PA-Ni was tested by Fourier transform infrared (FTIR) spectroscopy (Thermo Scientific IS5), and the test range was 4000 ~ 500 cm^−1^. The thermal stability of different RPUFs under N_2_ conditions was tested using the DTG-60AH thermogravimetric analyzer (TGA, Shimadzu). The temperature range was 40–800 °C, and the heating rate was 5, 10, 20, 30 and 40 °C/min, respectively. The mass of the sample was 3–5 mg. The limiting oxygen index (LOI) value of the RPUF was recorded using the FTT-1402072 (FTT, UK). According to ISO 5660 standard, the flame retardant performance was investigated by using the FTT-CONE-0242 cone calorimeter (CONE, FTT). The test time was 600 s and the sample size was 100 mm × 100 mm × 10 mm. In order to simulate the fire scene with low fire radiation intensity in the initial stage of smoldering fire, the thermal radiation flux of the cone calorimeter was concentrated in the low radiation region, and the thermal radiation flux 25 and 35 kW/m^2^ were thus selected in this study. In addition, 50 kW/m^2^ will be used to test flashover phenomena in simulated fires fields. Scanning electron microscopy (SEM, Thermo Fisher Scientific, USA) was used to observe the microscopic residual carbon morphology of the RPUF after CONE combustion. The smoke density (Ds) and light transmittance of different RPUFs in flame and flameless states were measured by NBS smoke density chamber (FTT) during 1200 s. The sample size was 70 mm × 70 mm × 10 mm. According to the ISO 845–2006, the apparent capacity of the sample was calculated by the mass-volume method, and the product mass was weighed on the analytical balance, and the sample specification was 50 mm × 50 mm × 50 mm. The compression-resistant test was carried out on the microcomputer-controlled electronic universal testing machine (GTM, China), the setting speed was 5.0 mm/min, and the specimen specifications were 50 mm × 50 mm × 50 mm.

### Theoretical approach

#### IPDT

Integral programmed decomposition temperature (IPDT)^15^ is an important parameter to evaluate the thermal stability of polymers, and as shown in Eqs. (1)–(3):

1 $$IPDT = AK(T_{f} - T_{i} ) + T_{i}$$

2 $$A = (S1 + S2)/(S1 + S2 + S3)$$

3 $$K = (S1 + S2)/S1$$

In this method, *A* is the area ratio of the different weightless areas on the TG curve, *K* is the scale factor, and *T*_*i*_ and *T*_*f*_ are the initial temperature and final temperature in the thermogravimetric analysis experiment.

#### Pyrolysis kinetics

Previous studies have given a detailed kinetic derivation of the process^16^, based on the Flynn–Wall–Ozawa (FWO)^17^ method expressed as shown in Eq. (4):

4 $$\lg \beta = \lg \frac{AE}{{G(\alpha )R}} - 2.315 - \frac{0.4567E}{{RT}}$$

The *α* values of different conversion rates are obtained by linear fitting of lg*β* and 1/*T*, where *β* is the heating rate and R is the constant.

Compared with FWO, the activation energy (E) obtained by the Starink^18^ method is more accurate, and as shown in Eq. (5):

5 $${\text{ln}}\frac{\beta }{{T^{1.92} }} = Cs - \frac{BE}{{RT}}$$

where B = 1.0008.

Different from the first two methods, in order to better evaluate the E during the pyrolysis process, the E of the maximum thermal decomposition rate obtained by the Kissinger^19^ method can be expressed by Eq. (6):

6 $$\ln \left( {\frac{{\beta_{i} }}{{T_{pi}^{2} }}} \right) = \ln \frac{{A_{k} R}}{{E_{k} }} - \frac{{E_{k} }}{R}\frac{1}{{T_{pi} }}\;{\text{i}} = (1,2,3,4)$$

*β*_*i*_ is the heating rate, and *T*_*pi*_ is the maximum thermal decomposition rate temperature.

According to the Kissinger method, Coats–Redfern (C–R)^20^ can provide E in different stages, as shown in Eq. (7):

7 $$\ln \frac{g(\alpha )}{{T^{2} }} = \ln \left[ {\frac{AR}{{\beta E}}\left( {1 - \frac{2RT}{E}} \right)} \right] - \frac{E}{RT}$$

In the current work, four methods are combined to analyze the apparent E of the modified RPUFs.

#### Fire risk assessment index

The fire risk assessment index is given from the CONE data: Toxic Gas Production Index (ToxPI), The smoke production index (TSPI), Fire growth index (FGI) and The heat release index (THRI). The formulas are as follows Eqs. (8), (9), (10) and (11)^21^.

8 $$ToxPI = \lg (COY \times MLR \times 10^{3} )$$

9 $$TSPI = \lg (SEA \times MLR \times t_{s} \times 10^{ - 1} )$$

10 $$FGI = \frac{PHRR}{t}$$

11 $$THRI = \lg (HRR \times t_{s} \times 10^{ - 3} )$$

In these equations, the first 120 s of the CONE experiment were selected.

## Results and discussion

### Characterization of the flame retardant PA-Ni

**Figure 3:**
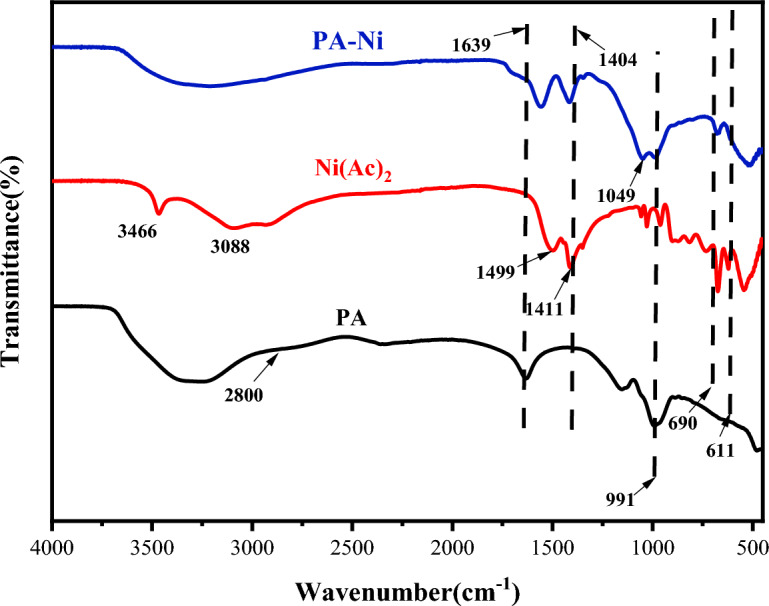
FTIR of PA, Ni(Ac)_2_ and PA-Ni.

Figure 3 displayed the FTIR spectra of PA, Ni(Ac)_2_ and PA-Ni. The distinctive absorption peak of (PO_3_)^2−^ was assigned a wavelength of 991 cm^−1^ in the PA spectrum. The stretching vibration of C–H in–CH_3_ for the Ni(Ac)_2_ spectrum was 3088 cm^−1^, whereas the bending vibration of C–H was 1411 cm^−1^. The typical O–H absorption peak of H_2_O was 3410 cm^−1^, the characteristic O–P–O absorption peak was 1639 cm^−1^, the characteristic P = O absorption peak was 1137 cm^−1^, the characteristic C–H stretching vibration peak was 2848–2940 cm^−1^^22^, and the characteristic P–OH absorption peak was 2800 cm^−1^^23^. The distinctive peaks of nickel salts were assigned to 611 cm^−1^ and 690 cm^−1^, whereas O=C and C–O were responsible for the absorption peaks of 1560 cm^−1^ and 1404 cm^−1^. The distinctive absorption peaks of (PO_3_)^2−^ migrated from 991 cm^−1^ to 1049 cm^−1^ in the PA-Ni spectra, while the characteristic absorption peaks of nickel salts were 611 cm^−1^ and 690 cm^−1^. In addition, FTIR spectra of PA-Ni compared to PA and Ni(Ac)_2_, the disappearance of the characteristic absorption peak of P–OH at 2800 cm^−1^ and the disappearance of the tensile vibration of –CH_3_ at 3088 cm^−1^ also indicated the successful synthesis of PA-Ni.

### Thermal stability

#### TG analysis

Thermogravimetric analysis (TGA) is a thermal analysis technique. The change in sample mass with temperature is measured at a programmed controlled temperature^24^. The reaction mechanism is that as the specimen reacts in different ways, a mass change occurs, resulting in steps on the TGA curve or peaks on the DTG curve, which can be used to study the thermal stability of the material. In practical materials analysis, TGA is often used in conjunction with other analytical methods for a comprehensive thermal analysis. Five heating rates were chosen in the current work. This allows for a better study of the pyrolysis behaviour of the samples under different conditions. Also, the use of five heating rates allows for the calculation of activation energy (E), which improves the accuracy of E and better analyses the thermal stability of the samples.

**Figure 4:**
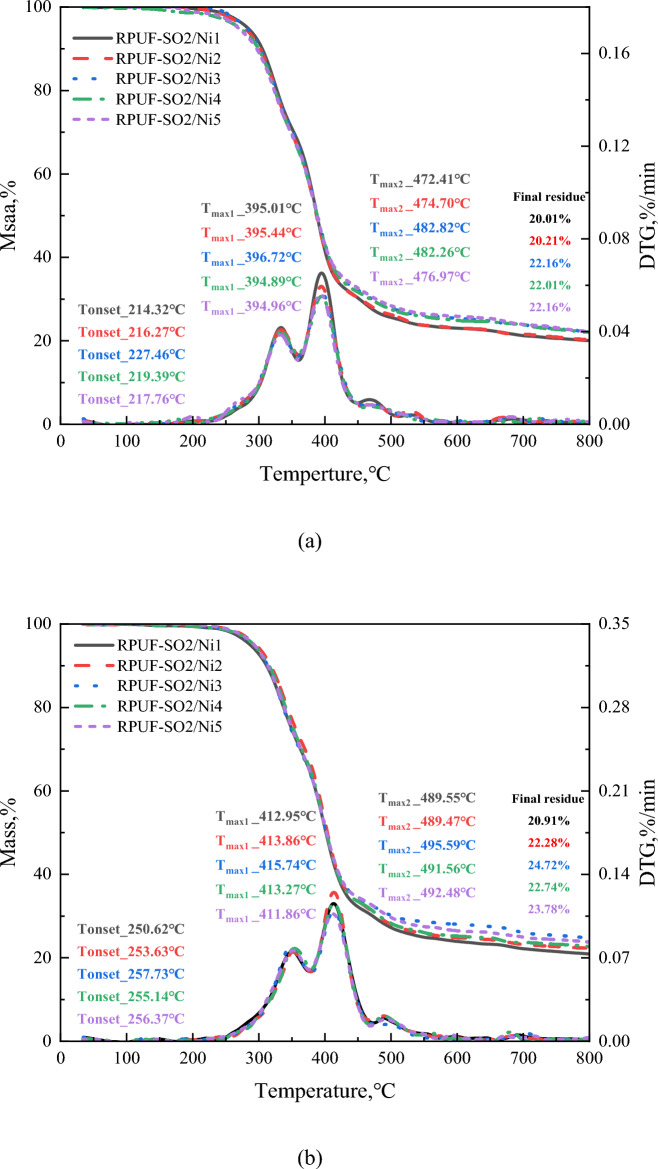

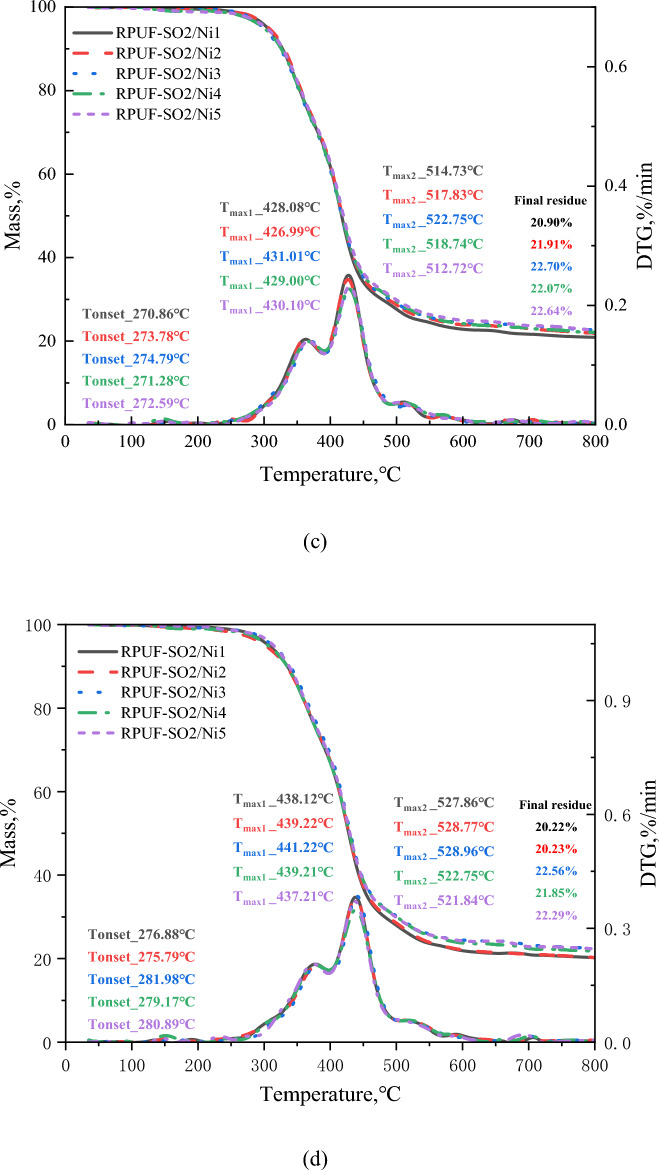

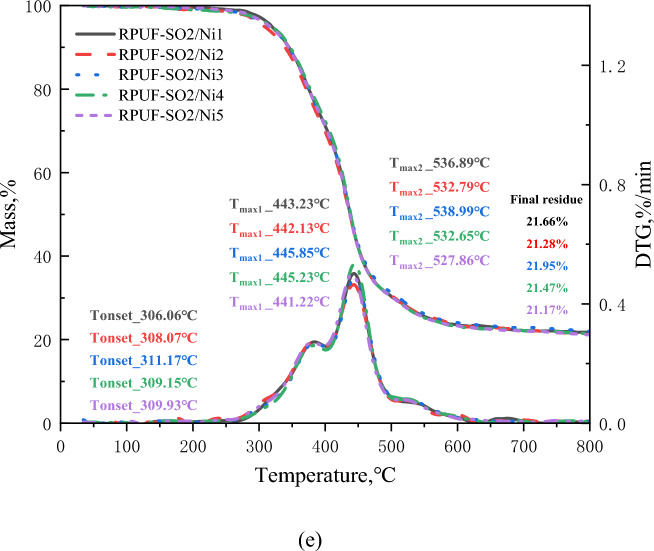
TG and DTG curves of the modified RPUFs at different heating rates. 5 °C/min (**a**), 10 °C/min (**b**), 20 °C/min (**c**), 30 °C/min (**d**) and 40 °C/min (**e**).

Both TGA and differential thermogravimetric (DTG) were important tools for assessing the thermal stability of polymer materials. The TG and DTG curves for all materials at different heating rates were shown in Fig. 4, and Table 2 showed the important parameters of the RPUFs in terms of thermal stability. As can be seen from Fig. 4a, the pyrolysis of the modified RPUF was divided into three regions, which was also the same reaction mechanism and weight loss stage as for the samples without added PA-Ni^31^. For RPUF-SO2/Ni3, the first region of weight loss was between 227.46 and 445.23 °C. The weight loss was approximately 66.76%, which was caused by the decomposition of the hard segments of the RPUF molecular chain. And the thermal decomposition of PA-Ni produced small molecules of phosphoric acid and pyrophosphate, which was also accompanied by the formation of nickel phosphate salts^14^. The macromolecular chain decomposition of SO polyols also released some non-flammable gases, such as CO_2_ and H_2_O^31^. It was clear from the DTG curve in Fig. 4a that the maximum decomposition rate temperature of RPUF-SO2/Ni3 (T_max1_, 396.72 °C) was the highest, which was due to the decomposition of PA-Ni that produced cross-linked phosphorus oxide and nickel oxides. In addition, the introduction of SO polyols improved the degree of cross-linking of the RPUF matrix, and the two synergistically improved the thermal stability of the RPUF matrix, so the higher temperatures were required for decomposition. The second area of weight loss was between 445.23 and 570.37 °C. The weight loss was about 7.04%, and this region corresponds to the decomposition of the soft segments of the RPUF molecular chain^24^. The temperature region of the last weightlessness was at 570.37–800.00 °C, and the weightlessness rate was about 3.62%, which was the further decomposition of the residue and finally the quality remains stable. In addition, the RPUF-SO2/Ni3 residual rate (R_%_) was shown in Fig. 4a, and the initial decomposition temperature (T_I_) and termination temperature (T_T_) were listed in Table 2. It can be clearly seen that at 5 °C/min, the R_%_ of RPUF-SO2/Ni1, RPUF-SO2/Ni2, RPUF-SO2/Ni3, RPUF-SO2/Ni4 and RPUF-SO2/Ni5 were 22.01%, 22.21%, 22.16%, 22.01% and 22.16%, respectively, compared with RPUF-SO2 (18.90%), the residual amount of the modified material was increased, and the residual amount of RPUF-SO2/Ni3 increased the most, by 3.26%. In terms of T_I_ and T_T_, compared with RPUF-SO2 (206.54 and 558.38 °C), the T_I_ and T_T_ of the five composites were increased, and the T_I_ and T_T_ of RPUF-SO2/Ni3 (227.46 and 570.43 °C) increased by 20.92 °C and 12.05 °C, respectively. The above data can prove that RPUF-SO2/Ni3 had the best thermal stability.

**Table 2:** Typical pyrolysis data of the modified RPUFs.

Heating rate (°C/min)	Sample	Weight loss temperature range (°C)	Percent weightlessness (%)	Initial decomposition temperature (°C)	Termination temperature (°C)	IPDT (°C)
5	RPUF-SO2/Ni1	214.32–444.14	67.99	214.32	561.06	702.69
		444.14–561.06	7.81			
		561.06–800.00	3.45			
	RPUF-SO2/Ni2	216.27–445.26	68.47	216.27	562.71	706.24
		445.26–562.71	6.88			
		562.71–800.00	3.70			
	RPUF-SO2/Ni3	227.46–445.23	66.76	227.46	570.37	750.28
		445.23–570.37	7.04			
		570.37–800.00	3.62			
	RPUF-SO2/Ni4	219.39–443.21	65.85	219.39	569.89	744.42
		443.21–569.89	7.33			
		569.89–800.00	3.00			
	RPUF-SO2/Ni5	217.76–445.59	65.36	217.76	562.27	749.42
		444.59–562.27	6.88			
		562.27–800.00	4.10			
10	RPUF-SO2/Ni1	250.62–468.40	68.00	250.62	590.24	727.02
		468.40–590.24	6.25			
		590.24–800.00	3.17			
	RPUF-SO2/Ni2	253.63–468.79	67.54	253.63	591.76	758.29
		468.79–591.76	6.57			
		591.76.800.00	2.73			
	RPUF-SO2/Ni3	257.73–470.41	65.82	257.73	593.58	813.59
		470.41–593.58	4.07			
		593.58–800.00	3.41			
	RPUF-SO2/Ni4	255.14–469.22	66.75	255.4	589.16	767.12
		469.22–589.16	6.43			
		589.16–800.00	2.41			
	RPUF-SO2/Ni5	256.37–470.89	65.96	256.37	591.83	791.64
		470.89–591.83	6.11			
		591.83–800.00	2.79			
20	RPUF-SO2/Ni1	270.86–489.56	69.12	270.86	599.36	739.25
		489.56–599.36	6.26			
		599.36–800.00	1.90			
	RPUF-SO2/Ni2	273.78–490.47	68.65	273.78	600.96	755.92
		490.47–600.96	5.63			
		600.96–800.00	2.00			
	RPUF-SO2/Ni3	274.79–492.47	68.48	274.79	601.60	771.59
		492.47–601.60	5.63			
		601.60–800.00	1.52			
	RPUF-SO2/Ni4	271.28–490.42	66.60	271.28	598.06	758.37
		490.42–598.06	6.57			
		598.06–800.00	2.15			
	RPUF-SO2/Ni5	272.59–491.94	66.58	272.59	599.98	769.94
		491.94–599.98	5.81			
		599.98–800.00	2.51			
30	RPUF-SO2/Ni1	276.88–496.67	69.41	276.88	625.63	724.20
		496.67–625.63	8.50			
		625.63–800.00	0.89			
	RPUF-SO2/Ni2	275.79–497.58	68.93	275.79	626.32	724.48
		497.58–626.32	6.26			
		626.32–800.00	1.50			
	RPUF-SO2/Ni3	281.98–499.67	68.03	281.98	629.11	774.76
		499.67–629.11	5.63			
		629.11–800.00	1.66			
	RPUF-SO2/Ni4	279.17–497.32	67.69	279.17	627.19	758.15
		497.32–627.19	6.39			
		627.19–800.00	1.61			
	RPUF-SO2/Ni5	280.89–498.10	67.72	280.89	626.57	768.71
		498.10–626.57	5.77			
		626.57–800.00	2.10			
40	RPUF-SO2/Ni1	306.06–508.71	67.26	306.06	640.95	759.42
		508.71–640.95	6.39			
		640.95–800.00	1.32			
	RPUF-SO2/Ni2	308.07–506.70	65.68	308.07	641.31	750.49
		506.70–641.31	6.71			
		641.31–800.00	1.38			
	RPUF-SO2/Ni3	311.17–509.62	65.82	311.17	645.52	765.67
		509.62–645.52	6.7			
		645.52–800.00	1.51			
	RPUF-SO2/Ni4	309.15–504.69	65.82	30.69	643.67	755.41
		504.69–643.67	7.35			
		643.67–800.00	1.03			
	RPUF-SO2/Ni5	309.93–502.69	65.50	309.93	641.78	749.38
		502.69–641.78	7.52			
		641.78–800.00	1.33			

The data in Fig. 4b–e and Table 2 showed that at 10, 20, 30 and 40 °C/min, the T_max1_ of RPUF-SO2/Ni3 was 415.74 °C, 431.01 °C, 441.22 °C and 445.85 °C, the T_I_ was 257.73 °C, 274.79 °C, 281.98 °C and 311.17 °C, and the T_T_ was 593.58 °C, 601.60 °C, 629.11 °C and 645.52 °C, and R_%_ was 24.72%, 22.70%, 22.56% and 21.95%, respectively. The results showed that the T_max1_, T_I_, T_T_ and R_%_ of RPUF-SO2/Ni3 were the highest among all modifications. Compare with RPUF-SO2^31^, their T_max1_ increased by 4.82 °C, 5.47 °C, 5.98 °C and 6.24 °C, the T_I_ increased by 13.05 °C, 11.97 °C, 21.36 °C and 7.02 °C, the T_T_ increased by 7.42 °C, 22.10 °C, 7.45 °C and 8.62 °C, respectively. And the R_%_ also increased by 5.09%, 4.40%, 4.53% and 4.64%, respectively. The above data indicated that the introduction of PA-Ni had greatly improved the thermal stability of the modified RPUF, which can not only improve the stability of the internal matrix of RPUF, but also produce a thicker carbon layer, inhibit the contact between the matrix and the external flame, and thus improve the stability of the polymer.

#### IPDT analysis

IPDT is also an important way to assess the thermal stability of materials^15^. The higher the value, the better the thermal stability of the material. The important parameters of the modified RPUFs for IPDI were listed in Table 2. It was clear from Table 2 that RPUF-SO2/Ni3 had the highest IPDT values of 750.28 °C, 813.59 °C, 771.59 °C, 774.76 °C and 765.67 °C under different conditions. Compared with the unmodified RPUF-SO2^31^, its results were increased by 74.48 °C, 114.12 °C, 86.97 °C, 93.35 °C and 76.99 °C, respectively. This further suggested that the thermal stability of the modified RPUFs was improved with the addition of PA-Ni. The results were in agreement with the TG study, which showed that RPUF-SO2/Ni3 had the best thermal stability.

### Thermodynamic analysis

The apparent activation energy (E) obtained by FWO and Starink methods were shown in Tables 3 and 4, and the relevant data of Kissinger and C-R method were shown in Tables 5 and 6. Table 3 showed that the average E values of RPUF-SO2/Ni1, RPUF-SO2/Ni2, RPUF-SO2/Ni3, RPUF-SO2/Ni4 and RPUF-SO2/Ni5 were 157.46, 158.80, 160.09, 156.31 and 158.16 kJ/mol, respectively. The mean E value of RPUF-SO2/Ni3 was the highest, which increased by 3.61 kJ/mol compared with that of RPUF-SO2 (156.48 kJ/mol) in the control group. The results in Table 4 also indicated that RPUF-SO2/Ni3 (157.83 kJ/mol) had the highest average E value of all RPUFs. Compared with RPUF-SO2 (153.55 kJ/mol), the E of RPUF-SO2/Ni3 increased by 4.28 kJ/mol. It can be seen that there were differences in the apparent E of different *α*, which also leaded to a significant change in the *E* value of different modified RPUFs with *α*, which was mainly due to the thermal unstable bonds of the materials and the different heating rates. The data in Table 5 showed that the E values of the modified RPUFs were 148.49, 165.01, 181.09, 173.18 and 179.34 kJ/mol, respectively. And the E of RPUF-SO2/Ni3 was the highest among all RPUFs, and its E was increased by 5.85 kJ/mol compared with the untreated RPUF-SO2 (175.24 kJ/mol). Table 6 concluded that RPUF-SO2/Ni3 had the highest E values in the three decomposition stages at five heating rates, which were 94.84, 99.96, 98.23, 86.84 and 85.45 kJ/mol, respectively. Compared with RPUF-SO2^31^, the E of RPUF-SO2/Ni3 increased by 2.11, 3.34, 5.61, 4.3 and 5.71 kJ/mol, respectively. It can be found that the results of the four calculation methods were the same, and RPUF-SO2/Ni3 had the best thermal stability, which was also consistent with the results of TG and IPDT analysis. This was mainly due to the fact that the introduction of PA-Ni enhanced the stability of the chemical bonds of the molecular chains within the modified RPUFs, which leaded to the fact that more heat was required to break the O=C and C–O and other chemical bonds within the RPUF during the thermal decomposition process, thus increasing the apparent E of RPUF and enhancing the thermal stability of the RPUFs.

**Table 3:** Calculated E of the modified RPUFs at different conversion rates with the FWO method.

α	RPUF-SO2/Ni1 E (kJ/mol)	RPUF-SO2/Ni2 E (kJ/mol)	RPUF-SO2/Ni3 E (kJ/mol)	RPUF-SO2/Ni4 E (kJ/mol)	RPUF-SO2/Ni5 E (kJ/mol)
10	143.05	142.80	144.39	129.72	120.85
20	132.70	146.49	134.44	135.46	126.08
30	126.09	135.74	128.35	127.16	121.96
40	138.62	140.84	137.04	129.80	131.14
50	149.51	153.62	149.32	144.87	145.32
60	154.14	156.00	157.82	155.46	153.99
70	156.66	158.40	163.89	162.26	162.42
80	168.24	166.88	173.87	172.70	183.12
90	248.11	228.45	251.68	249.39	278.59
E	157.46	158.80	160.09	156.31	158.16

**Table 4:** Calculated E of the modified RPUFs at different conversion rates with the Starink method.

α	RPUF-SO2/Ni1 E (kJ/mol)	RPUF-SO2/Ni2 E (kJ/mol)	RPUF-SO2/Ni3 E (kJ/mol)	RPUF-SO2/Ni4 E (kJ/mol)	RPUF-SO2/Ni5 E (kJ/mol)
10	144.73	159.86	142.37	143.25	120.87
20	133.46	144.10	131.53	141.68	144.78
30	126.22	132.58	124.84	132.58	154.80
40	130.72	137.59	133.69	137.59	138.15
50	141.90	150.73	146.25	150.73	135.25
60	154.90	153.05	155.01	153.05	144.38
70	157.39	155.38	161.21	149.57	153.77
80	169.29	164.02	171.41	155.72	169.51
90	244.10	211.23	254.18	227.82	249.19
E	155.86	156.50	157.83	154.67	156.74

**Table 5:** Calculated E of the modified RPUFs with the Kissinger device.

Stage	RPUF-SO2/Ni1 E (kJ/mol)	RPUF-SO2/Ni2 E (kJ/mol)	RPUF-SO2/Ni3 E (kJ/mol)	RPUF-SO2/Ni4 E (kJ/mol)	RPUF-SO2/Ni5 E (kJ/mol)
Slope K = − E/R	17,860.64	19,847.67	21,780.97	20,829.59	21,570.48
activation energy EK (kJ/mol)	148.49	165.01	181.09	173.18	179.34
Correlation coefficient R	0.99	0.99	0.99	0.99	0.99

**Table 6:** Calculated E of the modified RPUFs with the C-R method.

Heating rate β (°C/min)	Sample	Temperature range (°C)	E (kJ/mol)	E (kJ/mol)
5	RPUF-SO2/Ni1	214.32–444.14	71.93	93.76
		444.14–561.06	11.85	
		561.06–800.00	9.98	
	RPUF-SO2/Ni2	216.27–445.26	68.81	84.34
		445.26–562.71	7.58	
		562.71–800.00	7.95	
	RPUF-SO2/Ni3	227.46–445.23	65.36	94.87
		445.23–570.37	10.70	
		570.37–800.00	18.81	
	RPUF-SO2/Ni4	219.39–443.21	65.60	93.83
		443.21–569.89	9.48	
		569.89–800.00	18.75	
	RPUF-SO2/Ni5	217.76–445.59	59.66	76.99
		444.59–562.27	7.70	
		562.27–800.00	9.63	
10	RPUF-SO2/Ni1	250.62–468.40	70.89	91.16
		468.40–590.24	13.40	
		590.24–800.00	6.86	
	RPUF-SO2/Ni2	253.63–468.79	64.30	97.79
		468.79–591.76	18.83	
		591.76.800.00	14.66	
	RPUF-SO2/Ni3	257.73–470.41	69.46	99.96
		470.41–593.58	13.17	
		593.58–800.00	17.32	
	RPUF-SO2/Ni4	255.14–469.22	69.40	97.26
		469.22–589.16	19.13	
		589.16–800.00	8.73	
	RPUF-SO2/Ni5	256.37–470.89	69.07	97.57
		470.89–591.83	17.30	
		591.83–800.00	11.20	
20	RPUF-SO2/Ni1	270.86–489.56	61.27	95.46
		489.56–599.36	16.00	
		599.36–800.00	18.20	
	RPUF-SO2/Ni2	273.78–490.47	62.33	93.03
		490.47–600.96	16.44	
		600.96–800.00	14.27	
	RPUF-SO2/Ni3	274.79–492.47	62.92	98.23
		492.47–601.60	18.13	
		601.60–800.00	17.18	
	RPUF-SO2/Ni4	271.28–490.42	69.68	93.07
		490.42–598.06	16.91	
		598.06–800.00	6.48	
	RPUF-SO2/Ni5	272.59–491.94	72.63	92.97
		491.94–599.98	15.29	
		599.98–800.00	5.05	
30	RPUF-SO2/Ni1	276.88–496.67	65.58	82.47
		496.67–625.63	8.35	
		625.63–800.00	8.54	
	RPUF-SO2/Ni2	275.79–497.58	57.74	72.48
		497.58–626.32	10.44	
		626.32–800.00	4.30	
	RPUF-SO2/Ni3	281.98–499.67	66.54	86.84
		499.67–629.11	5.51	
		629.11–800.00	14.59	
	RPUF-SO2/Ni4	279.17–497.32	62.03	84.12
		497.32–627.19	12.05	
		627.19–800.00	10.05	
	RPUF-SO2/Ni5	280.89–498.10	72.36	82.88
		498.10–626.57	7.09	
		626.57–800.00	3.43	
40	RPUF-SO2/Ni1	306.06–508.71	72.71	84.63
		508.71–640.95	11.35	
		640.95–800.00	0.57	
	RPUF-SO2/Ni2	308.07–506.70	60.08	77.22
		506.70–641.31	12.05	
		641.31–800.00	5.09	
	RPUF-SO2/Ni3	311.17–509.62	65.50	85.45
		509.62–645.52	11.36	
		645.52–800.00	8.59	
	RPUF-SO2/Ni4	309.15–504.69	64.20	84.45
		504.69–643.67	14.08	
		643.67–800.00	6.16	
	RPUF-SO2/Ni5	309.93–502.69	66.25	80.30
		502.69–641.78	9.39	
		641.78–800.00	4.66	

### Flame retardancy of the modified RPUFs

**Figure 5:**
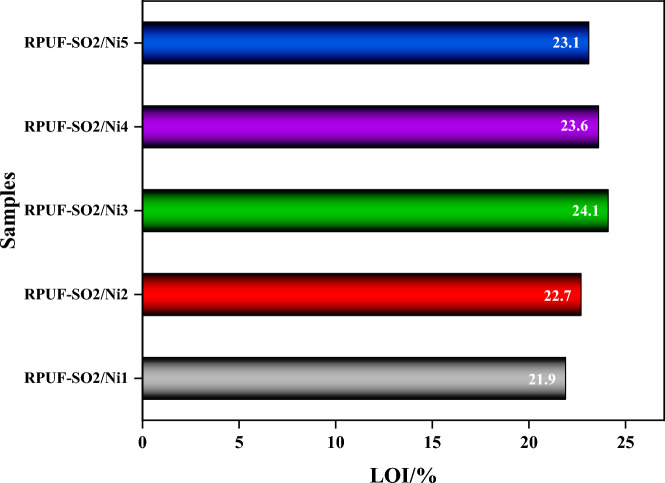
LOI of the modified RPUFs.

The LOI is used to evaluate the flame retardancy of the modified RPUFs, and the LOI values of the modified samples were shown in Fig. 5. It was found that when the amount of PA-Ni was gradually increased, the LOI of the RPUF firstly showed an increasing trend and then slightly decreased. Compared with the LOI of RPUF-SO2 (21.5%), the five RPUFs increased from 21.5% to 21.9%, 22.7%, 24.1%, 23.6% and 23.1%, respectively. It can be clearly seen that the introduction of PA-Ni can better improve the LOI of modified RPUF, among which RPUF-SO2/Ni3 had the best enhancement effect. The addition of PA-Ni to the original substrate of the RPUF can achieve better flame retardancy, which was due to the fact that PA-Ni produced some non-combustible nickel oxides and phosphoric acid derivatives attached to the porous carbon coke when it burned or decomposed, which effectively prevented the rupture of the carbon layer, and then formed a dense carbon layer to protect the internal substrate of the RPUF. At the same time, the modified RPUF contained P elements, which released reactive P-containing free radicals (PO· and PO_2_·) upon combustion. It can capture H·and HO· radicals in the gas phase, and had a quenching effect. In order to reactivate the activity of the free radicals, it was necessary to increase the O_2_ concentration, which leads to higher LOI values^25^. However, from the experimental results, although the LOI value of the modified RPUF was higher than that of RPUF-SO2. But, the LOI value decreased slightly with the excess addition of PA-Ni. This may be due to the fact that excessive PA-Ni generated internal stress during combustion expansion, which destroyed the integrity of the carbon layer and leaded to the rupture of the carbon layer, thus reducing the flame retardancy of the modified RPUF. Therefore, the addition of appropriate amount of PA-Ni had a better enhancement on the flame retardancy of the modified RPUF.

**Figure 6:**
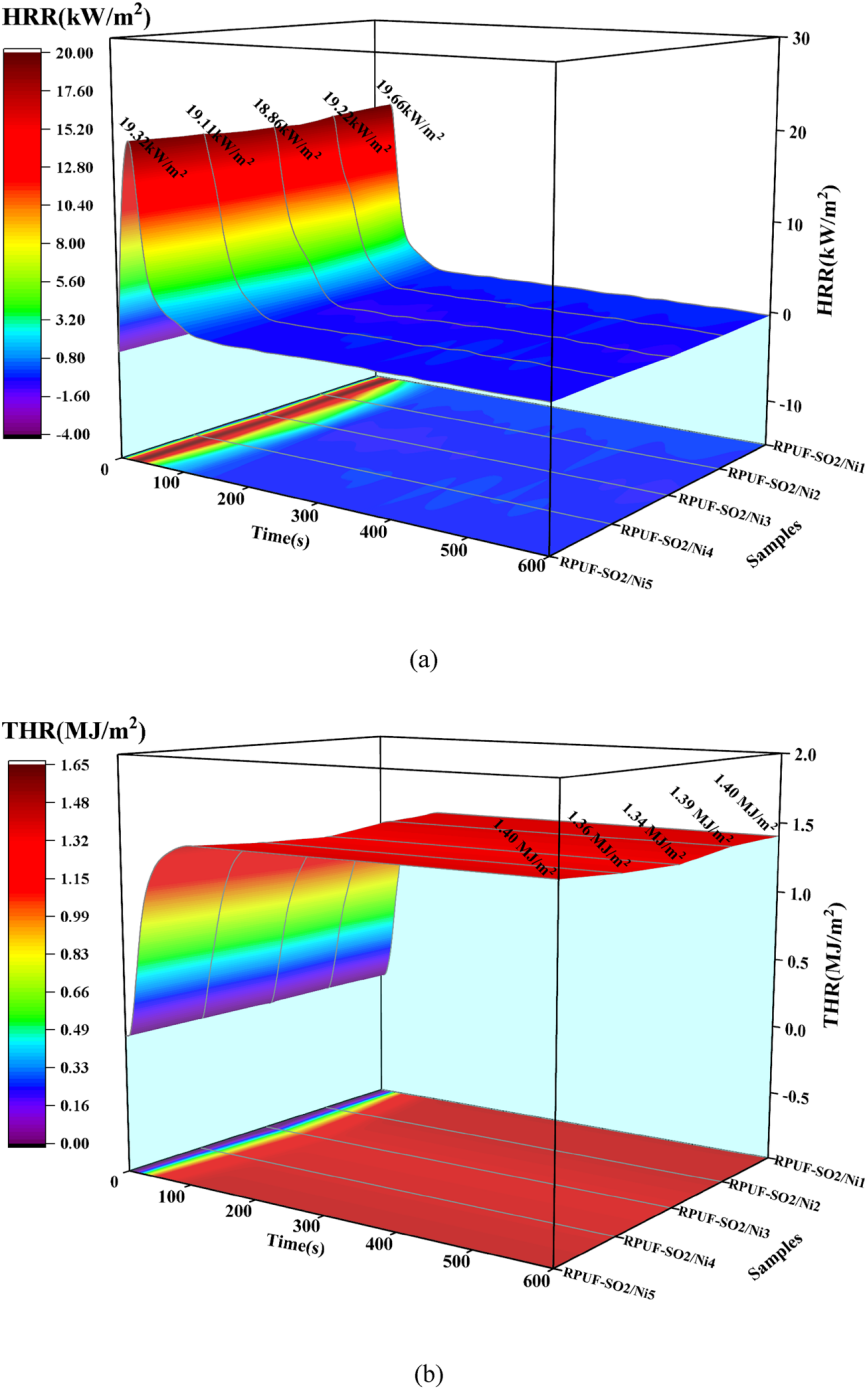

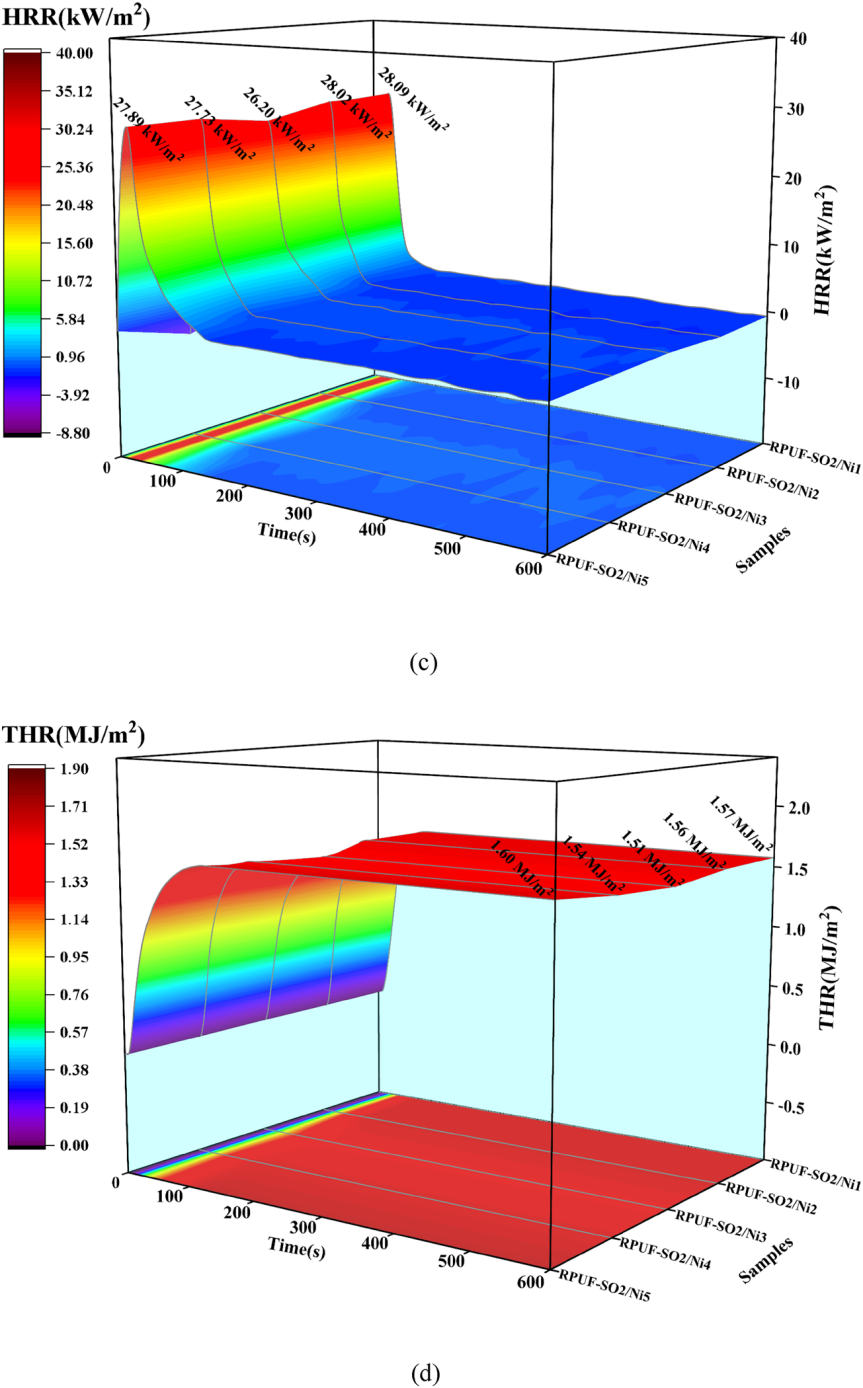

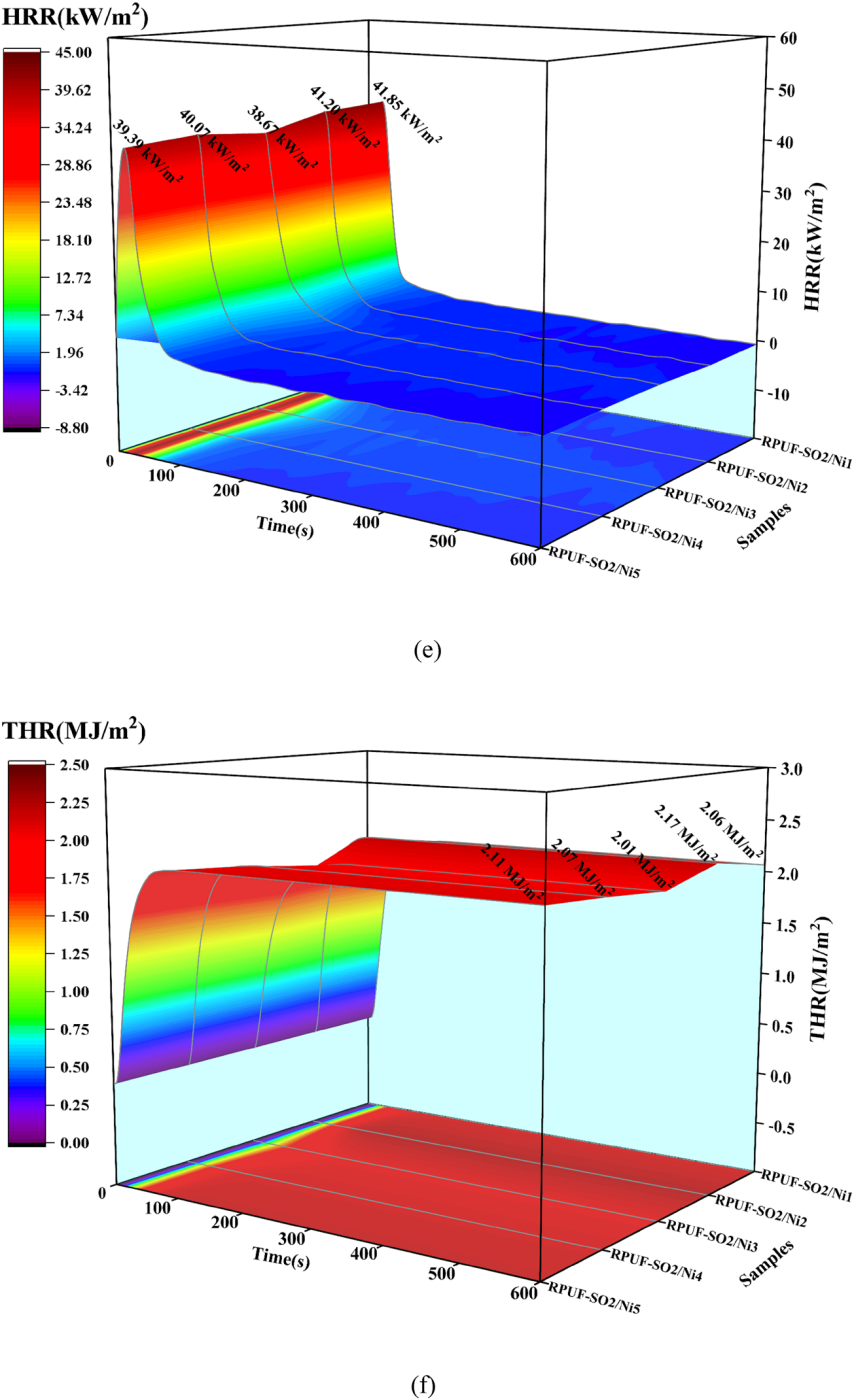
HRR and THR curves of the modified RPUFs at 25 kW/m^2^ (**a**,**b**), 35 kW/m^2^ (**c**,**d**) and 50 kW/m^2^ (**e**,**f**).

The flame retardancy of RPUFs was further investigated by CONE experiments, and the heat release rate (HRR) and total heat release rate (THR) curves of the modified RPUFs were shown in Fig. 6. At 50 kW/m^2^ (Fig. 6 e,f), the PHRR of RPUF-SO2/Ni1, RPUF-SO2/Ni2, RPUF-SO2/Ni2, RPUF-SO2/Ni3, RPUF-SO2/Ni4 and RPUF-SO2/Ni5 were 41.85, 41.20, 38.67, 40.07 and 39.39 kW/m^2^, respectively, compared with the PHRR of RPUF-SO2 (43.10 kW/m^2^), which decreased by 2.90%, 4.41%, 10.27%, 7.03% and 8.61%, respectively. The THR of the five modified samples was 2.06, 2.17, 2.01, 2.07 and 2.11 MJ/m^2^, respectively, and compared with the THR of RPUF-SO2 (2.51 MJ/m^2^), it was reduced by 17.92%, 13.55%, 19.92%, 17.53% and 15.94%, respectively. It was clear that the flame retardancy of the modified RPUFs with PA-Ni had been effectively enhanced. Among them, the PHRR and THR of RPUF-SO2/Ni3 were the lowest among all samples, and its flame retardant effect was the best. At the same time, it can be found that when the content of PA-Ni increased again, the flame retardant effect decreased, which was also consistent with the LOI analysis and once again proved that the appropriate amount of PA-Ni had the best flame retardant effect on the modified RPUFs. In addition, at 25 and 35 kW/m^2^, the PHRR of RPUF-SO2/Ni3 was 18.86 and 26.20 kW/m^2^, which was 0.42% and 9.62% lower than that of RPUF-SO2 (18.94 and 28.99 kW/m^2^), respectively. The THR of RPUF-SO2/Ni3 was 1.34 and 1.51 MJ/m^2^, which decreased by 4.96% and 6.21%, compared with RPUF-SO2 (1.41 and 1.61 MJ/m^2^). At these two radiant fluxes, RPUF-SO2/Ni3 still had the lowest PHRR and THR.

On the basis of the results of LOI and CONE experiments, the flame retardant performance can be greatly improved by adding an appropriate amount of PA-Ni to SO polyol-based RPUF. This was because SO polyols released non-combustible gases during thermal decomposition, while PA-Ni decomposed to produce nickel phosphate salts and nickel derivatives attached to the carbon network skeleton during combustion, thus protecting the denseness and integrity of the carbon layer and preventing the formation of cracks in the carbon layer. As a result, the two synergistically formed an insulating layer that protected the substrate and thus improved the fire resistance of the RPUF.

**Figure 7:**
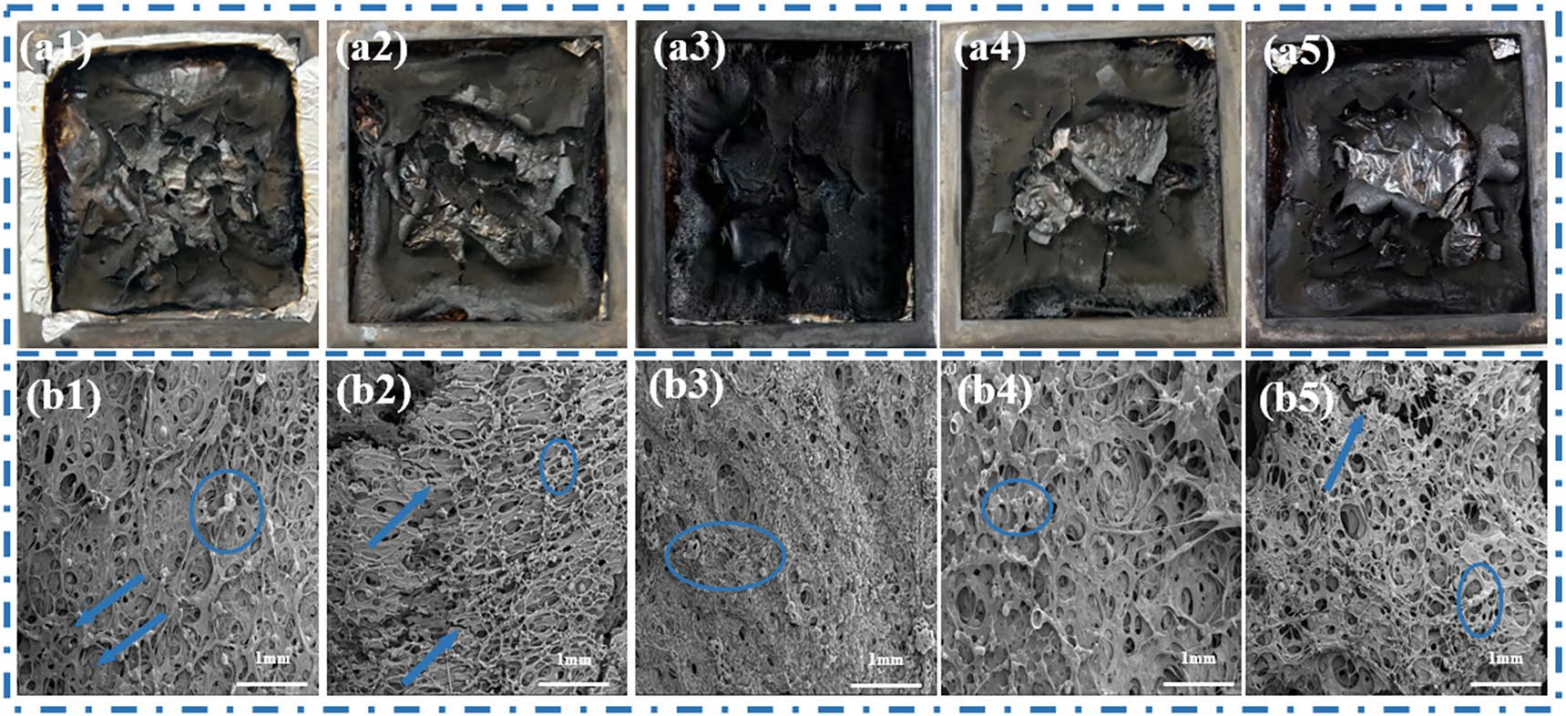
Macro and micro morphology of the modified RPUFs carbon residues after CONE test. RPUF-SO/Ni1 (**a1**,**b1**), RPUF-SO/Ni2 (**a2**,**b2**), RPUF-SO/Ni3 (**a3**,**b3**), RPUF-SO/Ni4 (**a4**,**b4**) and RPUF-SO/Ni5 (**a5**,**b5**).

The SEM of carbon residues of the treated specimen after CONE test was shown in Fig. 7. Figure 7 a (1–5) showed the original carbon residue, it was obvious that the carbon layer of the sample was very thin and had large cracks after combustion, among which RPUF-SO2/Ni1 had the largest cracking degree (Fig. 7a1). With the increase of PA-Ni, the degree of cracking gradually decreased in RPUF-SO2/Ni2 and RPUF-SO2/Ni3 (Fig. 7a2–a3). However, when PA-Ni was added again, the degree of cleavage of RPUF-SO2/Ni4 and RPUF-SO2/Ni5 increased again (Fig. 7a4–a5). In addition, the carbon layer of RPUF-SO2/Ni3 was the most intact, with fewer grooves and cracks, and less friable than the other samples^26^. From the SEM images of the residual carbon in Fig. 7b (1–5), it can be seen that the carbon layers of RPUF-SO2/Ni1 and RPUF-SO2/Ni2 were obviously cracked (Fig. 7b1–b2), which cannot prevent the external heat from entering into the matrix, and had poor flame retardant properties. However, RPUF-SO2/Ni3 was basically free of cracks (Fig. 7b3), and the charcoal mesh skeleton was more complete than the other materials. It also had relatively fewer pores and therefore the best flame retardancy, which was also consistent with the results of the macroscopic analyses. This was mainly due to the fact that the incorporation of PA-Ni and SO polyol improved the stability of the carbon network, and it can be found from the microstructure of the carbon layer that the phosphate derivatives and nickel oxides produced by the thermal decomposition of PA-Ni can be better connected with the carbon network in the carbon network skeleton^27^, which enhanced the compactness and integrity of the carbon layer, reduced the holes generation and inhibited the smoke spread. Therefore, the addition of PA-Ni can better improve the fire safety of the RPUF.

### Smoke analysis

**Figure 8:**
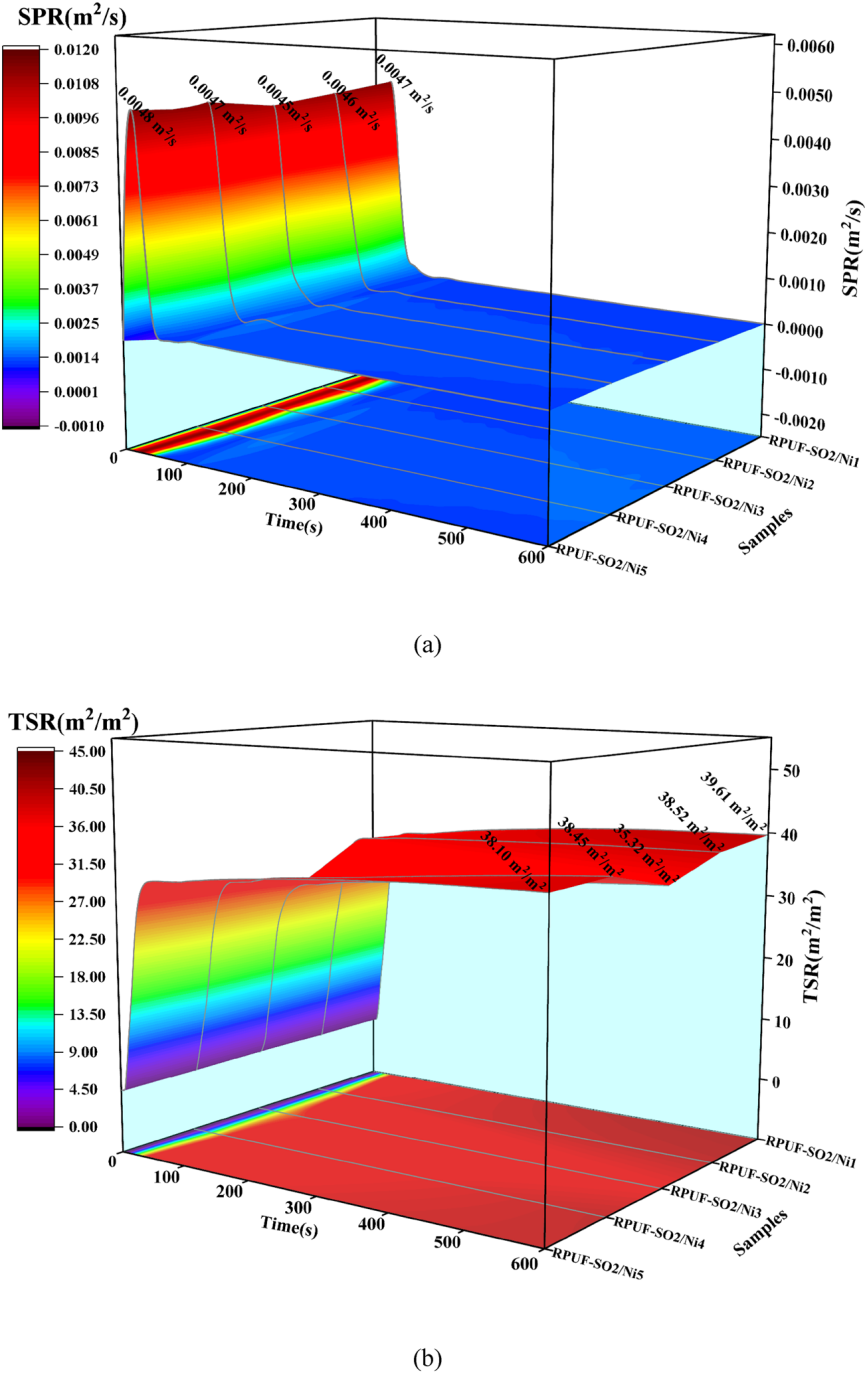

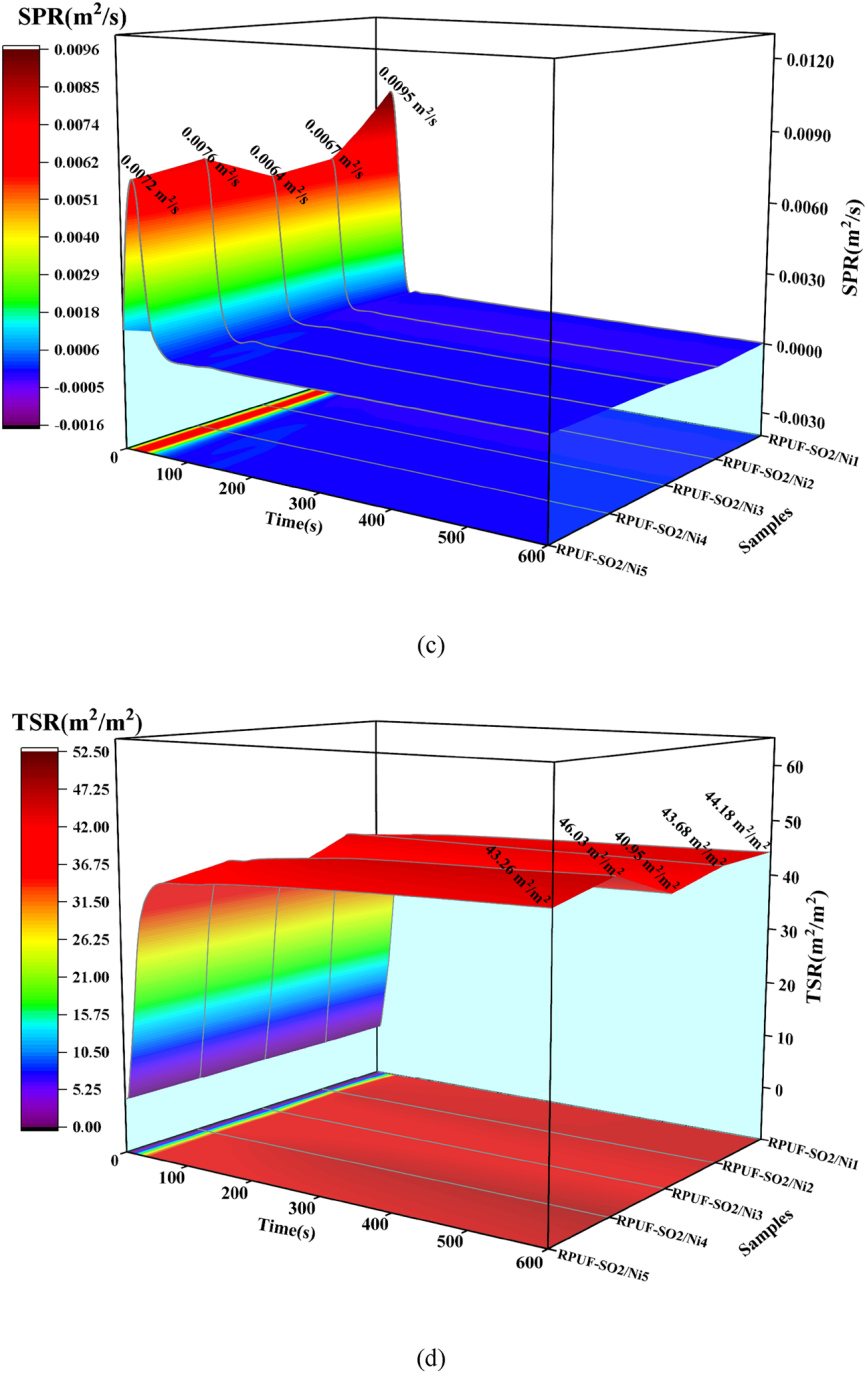

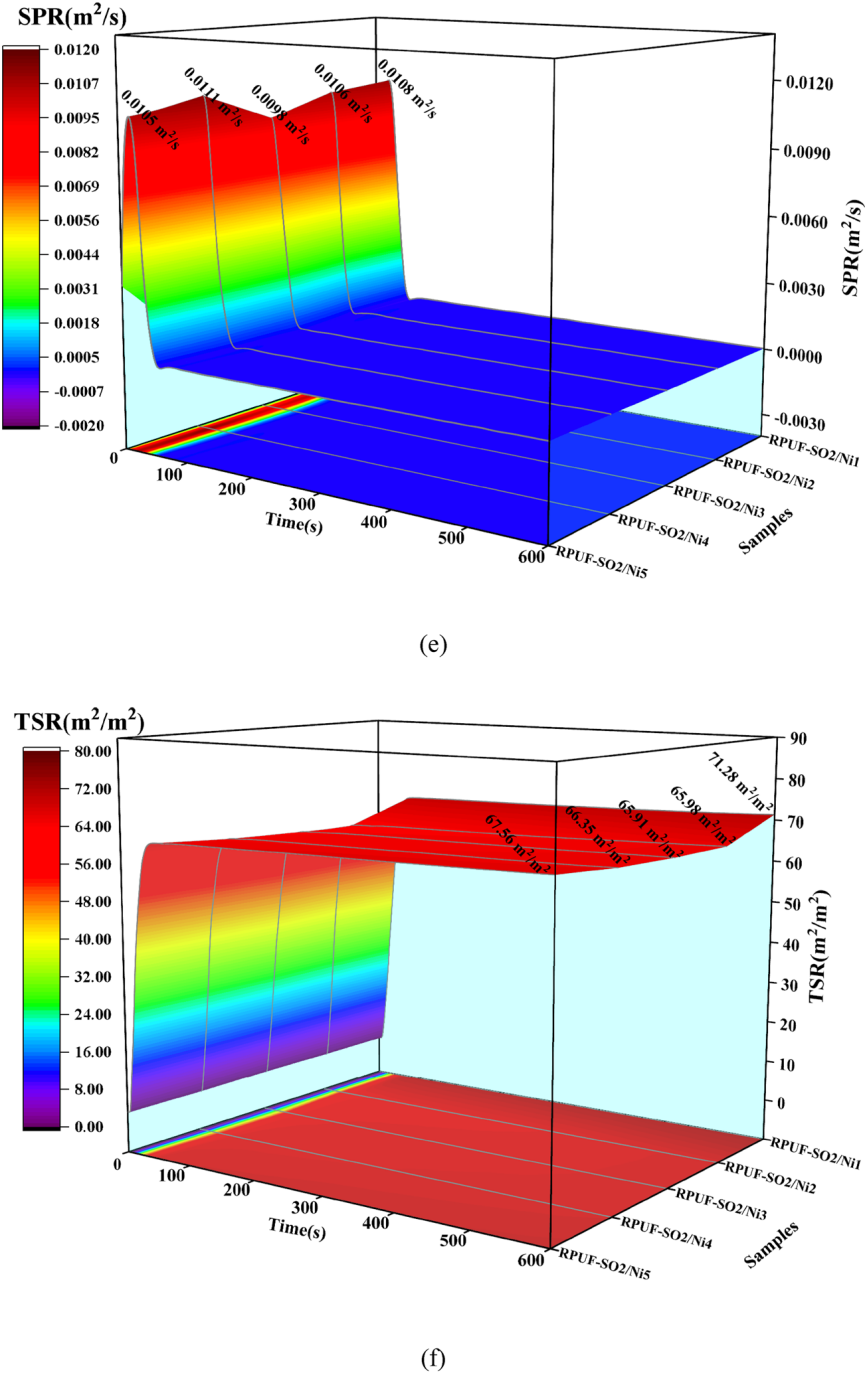
SPR and TSR curves of the modified RPUFs at 25 kW/m^2^ (**a**,**b**), 35 kW/m^2^ (**c**,**d**) and 50 kW/m^2^ (**e**,**f**).

Smoke is an important factor affecting people's lives during fire events^21^. The smoke production rate (SPR) and total smoke release (TSR) were shown in Fig. 8. Similarly, at 50 kW/m^2^ (Fig. 8e,f), when 1–3 wt% PA-Ni was added, it was clear that the SPR and TSR of the modified RPUFs gradually decreased, while the SPR and TSR of the samples increased again as the PA-Ni content increased. These data showed that RPUF-SO2/Ni3 had the lowest SPR (0.0098 m^2^/s) and TSR (65.91 m^2^/m^2^) and the best smoke suppression effect, which was also consistent with the results of HRR and THR analysis. Compared with the SPR (0.0116m^2^/s) and TSR (72.66m^2^/m^2^) of the unmodified RPUF-SO2, RPUF-SO2/Ni3 was reduced by 15.52% and 9.29%, respectively. At 25 kW/m^2^ and 35 kW/m^2^ (Fig. 8a–d), it can be found that the results were basically the same. At 25 kW/m^2^, the SPR (0.0045 m^2^/s) and TSR (35.32 m^2^/m^2^) of RPUF-SO2/Ni3 were the lowest, and compared with the SPR (0.0050 m^2^/s) and TSR (39.71 m^2^/m^2^) of RPUF-SO2, they were reduced by 10.00% and 12.29%, respectively. At 35 kW/m^2^, the SPR (0.0064 m^2^/s) and TSR (40.95 m^2^/m^2^) of RPUF-SO2/Ni3 were also the lowest, which were 16.88% and 8.94% lower than those of RPUF-SO2 (0.0077 m^2^/s) and TSR (44.97 m^2^/m^2^), respectively.

According to the above results, it can be found that the smoke-suppression effect of RPUF-SO2/Ni3 was the best. This is mainly due to the thermal decomposition of PA-Ni, which produced nickel phosphate and adhered to the porous carbon layer. At the same time, the presence of SO polyol increased the cross-linking degree of the modified RPUF, which promoted the production of a more stable and dense char layer during combustion. Together, they formed a fire barrier that inhibited the release of smoke and toxic gases. However, with the addition of excessive PA-Ni, the RPUF matrix was prone to agglomeration, resulting in excessive melt aggregation in the matrix, which leaded to the failure to improve the quality of the carbon layer during combustion^28^^.^ The gases produced by combustion broke through the poor-quality carbon layer. In turn, the carbon layer created a visible crack that cannot prevent the release of smoke, which was well supported by the results of SEM analysis. Therefore, the introduction of appropriate amount of PA-Ni can better inhibit the smoke release.

**Figure 9:**
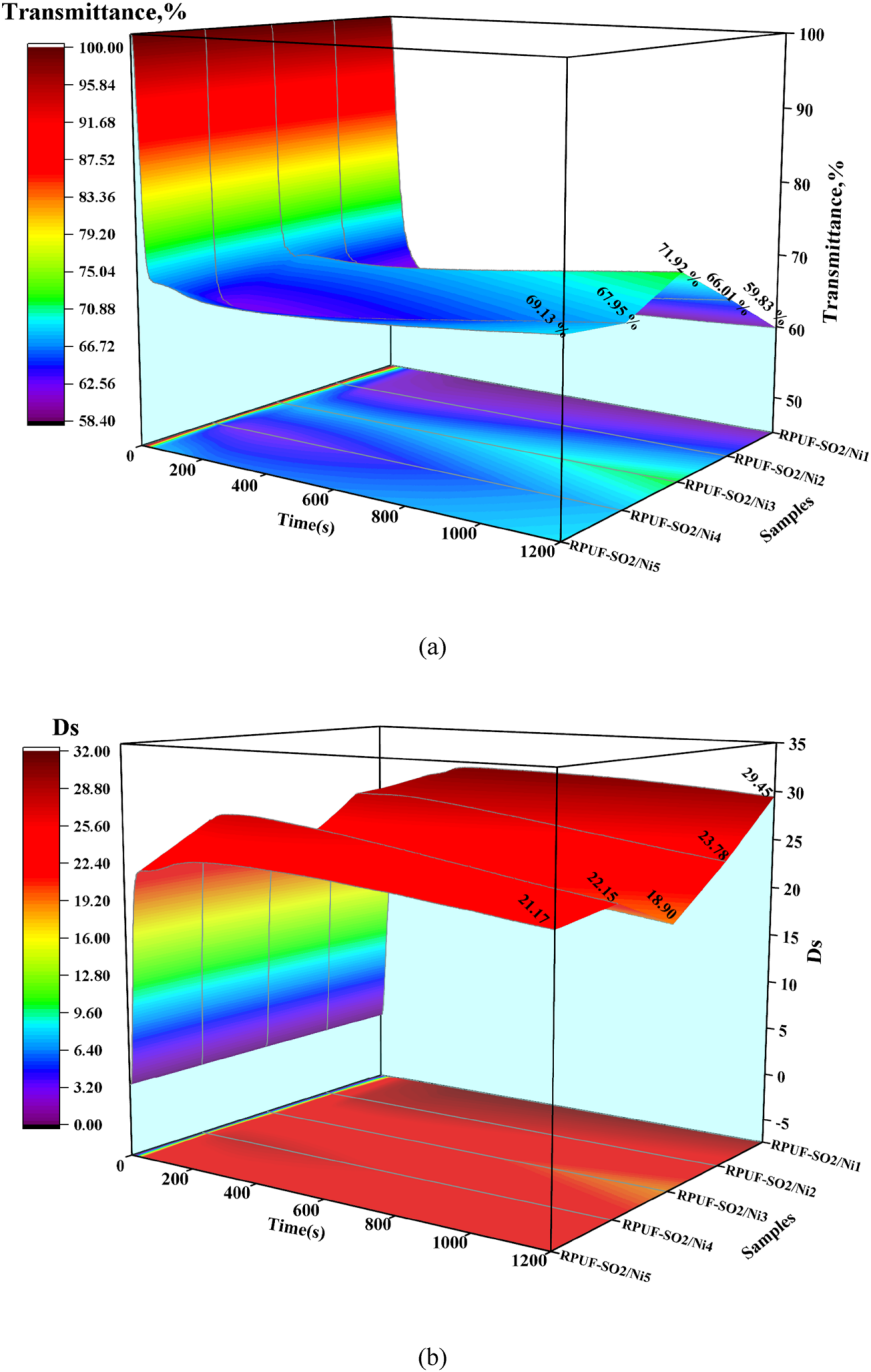

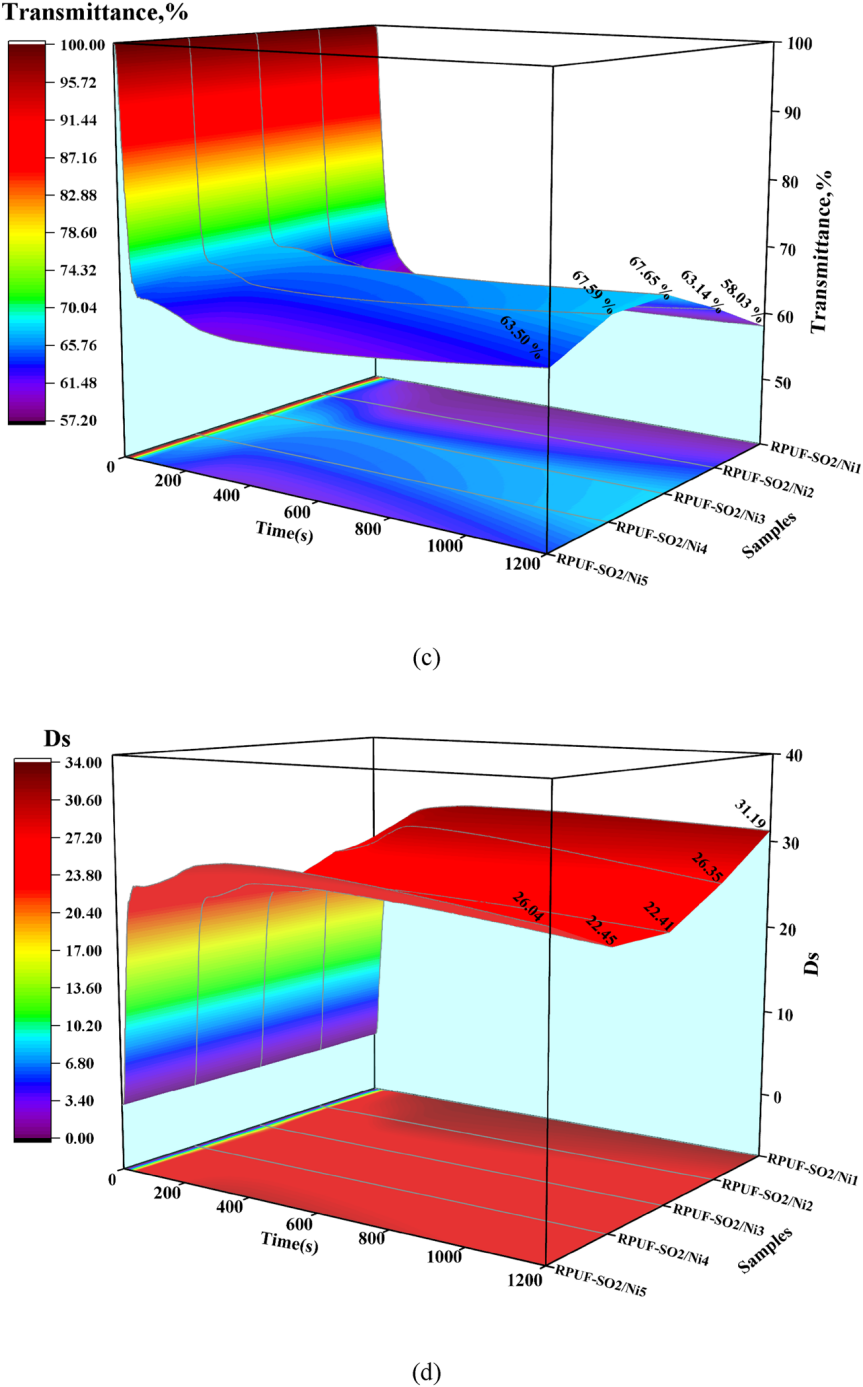
Transmittance and Ds curves of the modified RPUFs under flame conditions (**a**,**b**) and flameless conditions (**c**,**d**).

Ds is also a typical parameter for evaluating the smoke hazard of materials^25^. The light transmittance and Ds of the modified RPUFs under the flame and flameless conditions were shown in Fig. 9. Under the flame condition (Fig. 9a,b), the data showed that the Ds of RPUF-SO2/Ni3 was the smallest (18.90) and the transmittance (71.92%) was the largest, which was 41.77% lower than that of RPUF-SO2 (32.46) and 25.73% higher than that of RPUF-SO2 (57.20%). Under the flameless condition (Fig. 9c,d), the Ds of RPUF-SO2/Ni3 was 22.41 and the light transmittance was 67.65%, which was 22.91% lower than that of RPUF-SO2 (29.07) and 11.63% higher than that of RPUF-SO2 (60.60%). All the results indicated that RPUF-SO2/Ni3 had the best smoke suppression effect, which were consistent with the SPR and TSR results, and again demonstrated that the addition of PA-Ni was effective in improving the fire safety of RPUF.

### Fire hazard evaluation index

The fire hazard evaluation indexes under different conditions were listed in Table 7. At 25 kW/m^2^, it can be observed that the ToxPI, TSPI, FGI and THRI of RPUF-SO2/Ni3 were the lowest among all products, which were 0.21 kg/s, 2.18 m^2^/s, 0.75 kW/m^2^·s and 2.04 MJ/m^2^, respectively. ToxPI, TSPI, FGI and THRI were reduced by 32.26, 4.39, 9.63 and 24.16% respectively when compared to control RPUF-SO2^31^. At 25 and 50 kW/m^2^, the RPUF-SO2/Ni3 indexes were also the highest among all samples, and these indexes were reduced compared to the previously studied RPUF-SO2 composites^31^. This also corresponded to the Ds, HRR and THR analyses, further illustrating the lowest toxicity of RPUF-SO2/Ni3.

**Table 7:** Fire hazard evaluation indexes of the modified RPUFs under different conditions.

Radiation intensity (kW/m^2^)	Sample	ToxPI (kg/s)	TSPI (m^2^/s)	FGI (kW/m^2^·s)	THRI (MJ/m^2^)
25	RPUF-SO2/Ni1	0.26	2.22	0.77	2.16
	RPUF-SO2/Ni2	0.27	2.34	0.75	2.04
	RPUF-SO2/Ni3	0.21	2.18	0.61	2.01
	RPUF-SO2/Ni4	0.31	2.37	0.72	2.03
	RPUF-SO2/Ni5	0.39	2.39	0.81	2.14
35	RPUF-SO2/Ni1	0.43	2.44	1.21	2.27
	RPUF-SO2/Ni2	0.41	2.41	1.24	2.15
	RPUF-SO2/Ni3	0.33	2.35	1.16	2.10
	RPUF-SO2/Ni4	0.46	2.35	1.19	2.18
	RPUF-SO2/Ni5	0.37	2.48	1.24	2.16
50	RPUF-SO2/Ni1	0.38	2.43	1.77	2.22
	RPUF-SO2/Ni2	0.41	2.38	1.93	2.24
	RPUF-SO2/Ni3	0.31	2.30	1.65	2.23
	RPUF-SO2/Ni4	0.36	2.45	1.85	2.23
	RPUF-SO2/Ni5	0.35	2.51	1.82	2.31

### Mechanical properties analysis

**Figure 10:**
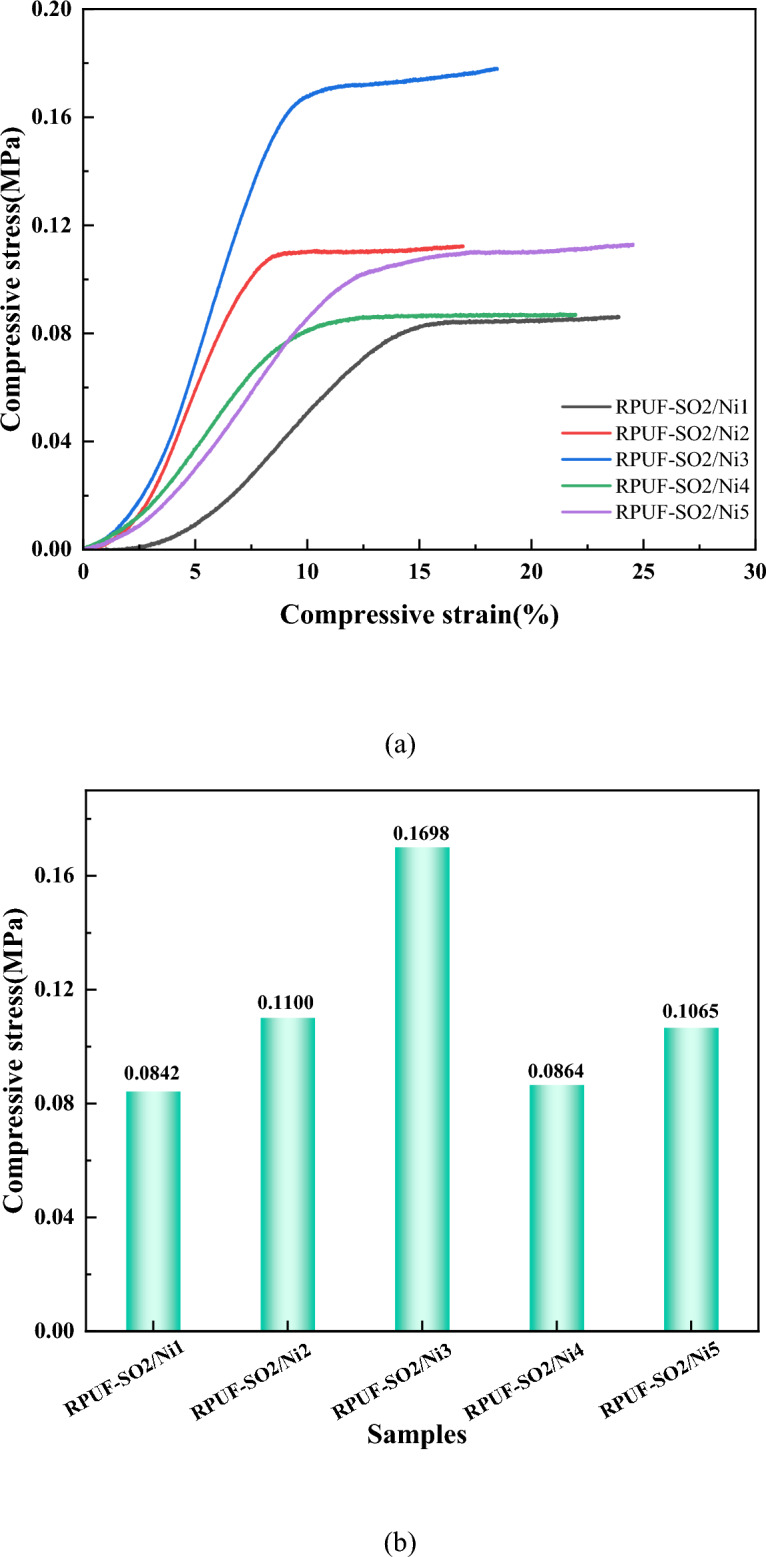
The stress–strain curves of the modified RPUFs and their compressive strength parameters Stress–strain (**a**) and Compressive strength (**b**).

Density and compressive strength are important factors affecting the mechanical properties of the RPUF^29^. The stress–strain curve of the modified RPUFs was shown in Fig. 10, and the relevant mechanical parameters and apparent density were listed in Table 8. Table 8 showed that the density of the modified RPUFs added with PA-Ni from 1 to 3 wt% increased gradually, from 32.49 to 53.35 kg/m^3^. When the PA-Ni content increased from 3 to 5 wt%, the apparent density decreased. It is worth noting that the compressive strength of the modified RPUFs was basically similar to the density law. When 1–3 wt% PA-Ni was added, the compressive strength increased from 0.0842 to 0.1698 MPa, and the compressive strength of RPUF-SO/Ni3 and RPUF-SO/Ni4 decreased by 0.0834 MPa and 0.1065 MPa with the increase of PA-Ni. This was because the higher content of PA-Ni destroyed the cell wall, resulting in a density decrease and thus reduced mechanical properties. In addition, the specific compressive strength and elastic modulus were also the key factors to judge the mechanical properties^31^. It can be seen that the specific compressive strength (3.18 MPa/(g/cm^3^)) and elastic modulus (0.0246 MPa) of RPUF-SO/Ni3 were the highest among all samples, which also proved that the mechanical properties of RPUF-SO/Ni3 were the best.

**Table 8:** Apparent density and mechanical properties of the modified RPUFs.

Samples	Apparent density (kg/m^3^)	Specific compressed strength [MPa/(g/cm^3^)]	Young's Modulus (MPa)
RPUF-SO2/Ni1	32.49	2.59	0.0093
RPUF-SO2/Ni2	38.15	2.88	0.0198
RPUF-SO2/Ni3	53.34	3.18	0.0264
RPUF-SO2/Ni4	34.98	2.49	0.0162
RPUF-SO2/Ni5	34.03	3.13	0.0120

### Flame retardant mechanism

**Figure 11:**
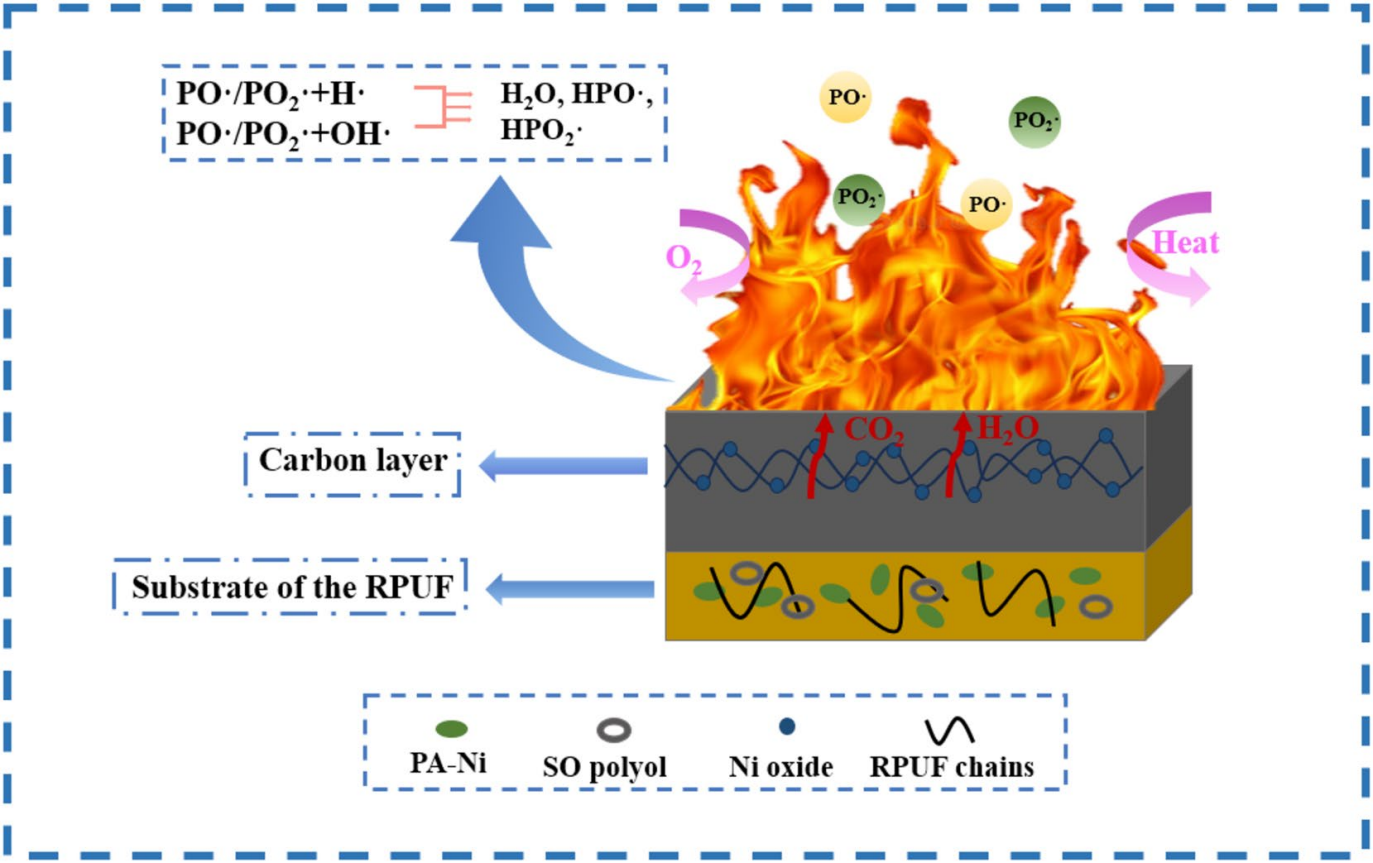
Schematic diagram of flame retardant mechanism of the modified RPUFs.

The mechanism of PA-Ni to improve the flame retardancy of the RPUF was shown in Fig. 11. Because PA-Ni itself contained P and Ni elements, PO· free radicals were released when the modified RPUF was burned, and H· free radicals and HO· free radicals were captured in the gas phase, the combustion of RPUF matrix can thus be inhibited^32^. At the same time, SO polyols will also release non-flammable gases such as H_2_O and CO_2_ when combusted, which reduced the concentration of O_2_ and combustible gases^15,33^. The synergistic effect of the two can better improve the flame retardant properties of the RPUF. Additionally, in the condensed phase, the nickel phosphate salt and nickel oxide produced by PA-Ni were attached to the carbon mesh framework, which improved the stability of the carbon layer and formed a denser and more complete carbon layer. It improved the fire safety of the modified RPUF by forming an insulating, flame-retardant outer thermal matrix and inhibiting the release of smoke and toxins.

## Conclusion

A novel biomass flame retardant, PA-Ni, was prepared and compounded with SO polyol for the flame retardant modification of the RPUF. The thermal stability, flame retardancy, smoke toxicity and mechanical properties of the modified RPUF were systematically investigated. The TG results showed that RPUF-SO2/Ni3 had excellent thermal stability, with the highest initial decomposition temperature, termination decomposition temperature, carbon residue, maximum thermal decomposition rate temperature, IPDT and E. And it also had the highest LOI of all RPUFs at 24.1%. Compared with RPUF-SO2, the PHRR of RPUF-SO2/Ni3 was reduced by 0.42%, 9.62% and 14.92%, and the THR was reduced by 4.96%, 6.21% and 19.92%, under 25, 35 and 50 kW/m^2^, which showed that RPUF-SO2/Ni3 had good flame retardancy. In addition, RPUF-SO2/Ni3 had the lowest SPR, TSR, Ds and the highest transmittance under flame and flameless conditions. At the same time, the fire hazard evaluation index pointed out that RPUF-SO2/Ni3 had the lowest ToxPI, TSPI, FGI and THRI, which further demonstrated that RPUF-SO2/Ni3 had the lowest smoke toxicity. It can be found that the apparent density of RPUF-SO2/Ni3 was the largest, and its compressive strength, specific compressive strength and elastic modulus were also the best among all RPUFs, which had better mechanical properties. Therefore, it can be concluded that the composite flame retardant system composed of PA-Ni can greatly improve the flame retardancy, smoke toxicity resistance and mechanical properties of the RPUF. The present explorations and results will open up new ideas for the green flame retardant modification of the RPUF, and also provide a good application prospect for the safe use of the RPUF.

## Data Availability

The data that support the findings of this study are available from the corresponding author upon reasonable request.
